# The short- and long-run effect of human capital on income inequality: Empirical evidence in the ASEAN region

**DOI:** 10.1371/journal.pone.0304678

**Published:** 2024-07-31

**Authors:** Duc Hong Vo, Anh The Vo, Chi Minh Ho

**Affiliations:** The Research Centre in Business, Economics and Resources, Ho Chi Minh City Open University, Ho Chi Minh, Vietnam; Islamia University of Bahawalpur, PAKISTAN

## Abstract

Human capital is a nation’s primary source of inner strength to achieve sustainable economic growth and development. Meanwhile, income inequality is a critical issue preventing sustainable economic growth and social transformation, especially in developing countries. This paper investigates the effect of human capital on income inequality in both the short and long term using the mean group, pooled mean group, and threshold regressions for the ASEAN-7 (including Indonesia, Laos, Malaysia, the Philippines, Singapore, Thailand, and Vietnam) from 1992 to 2018. The paper develops a theoretical linkage between human capital and income inequality by combining the learning theory and the Kuznets hypothesis. This linkage is then tested using data from the ASEAN countries. Findings from the paper indicate that human capital reduces income inequality in the short run in the ASEAN countries. However, the effect is reverted in the long run, suggesting that human capital may increase the income gap in these countries. Particularly, the inverted U-shaped relationship between human capital and income inequality is established for the ASEAN countries whose GDP per capita is lower than USD 8.2 thousand per year. In contrast, the U-shaped relationship is found for the countries with income per capital of more than USD 8.2 thousand. All these findings suggest that social policies targeting reducing income inequality should be prioritized and stay at the centre of any economic policies to achieve sustainable economic growth and development in the ASEAN countries.

## 1. Introduction

Since 2019, the term “4.0 industrial revolution” (or “digital revolution”) has been noticed intensively on various platforms such as newspapers, academic publications, development forums organized by global organizations (World Bank, United Nations) and government reports. According to the World Development Report by [1], in the Industrial Revolution 4.0, the global economy is changing and evolving toward the development of technology and the rise of digital platforms. Technology is changing labour demand from employers and pressuring employees to learn new skills such as complex problem-solving, teamwork and adaptability. Besides, the rapid development of digital technology is also changing how people work and the environment where they work. While the global market has been changing and adapting toward the 4.0 industrial revolution, the Association of Southeast Asian Nations (ASEAN) will face an ageing population in the next few decades [2]. Facing the upcoming challenges of the Industrial Revolution 4.0 and threats from ageing labour in the future, the governments in ASEAN have raised the importance of improving their human capital development for the ASEAN Community Vision 2025. In 2019, the development of human capital was also the major theme in the Sustainable Development Goals at the ASEAN High-Level Meeting on Human Capital Development [3].

After the global pandemic, the ASEAN has continued to recover at a relatively faster rate than the global economy, at 5.5 per cent compared to 3.5 per cent globally in 2022. Regardless of the steady economic growth rate, income inequality is still a critical concern for governments in the ASEAN region. According to [2], in the ASEAN region, limited education quality generates large learning gaps, and children have low reading comprehension skills at the end of primary school. Moreover, 15 per cent of 15-year-olds living today will not reach the age of 60 mainly due to noncommunicable diseases, namely diabetes, cancer, and cardiovascular disease. These issues result from unequal access to essential services caused by income inequality.

Income inequality has been perceived as the primary source of other inequality and poverty [4–12]. The problem of income inequality has also been emphasized as one of 17 sustainable development goals implied by the United Nations since 2016. The income gap causes divergence in the social hierarchy and worsens efforts to form an equalized society where all individuals should be treated fairly. Besides, the income gap favours advantaged groups to effectively defend and quickly adapt to economic and social shocks, while disadvantaged groups will be easily vulnerable to those changes.

Confronting the 4.0 industrial revolution and the depression after the global pandemic, enormous changes in the global economy, on the one hand, open great opportunities for individuals worldwide. Advantageous individuals have the resources to survive and adapt to pressure and changes from the coming revolution and the depression after the pandemic. On the other hand, these fluctuations in the current uncertain world have also created severe threats for poor people. Disadvantageous people face exposure to their jobs, poverty, and many other risks caused by the economic downturn after the COVID-19 pandemic and rapid changes in the market due to the 4.0 industrial revolution. As such, the ASEAN governments have urgently announced an appropriate and inclusive development framework in which all their civilians assess entirely basic needs, approach advanced knowledge, and embrace opportunities to enhance their productivity. Since then, the income gap between advantageous and disadvantageous groups has been narrowed down.

Theoretically, the Kuznets hypothesis emphasizes a trade-off between economic growth and income inequality in the former stage of industrialization. Then, the income gap will be reduced, and economic growth will occur later. From other perspectives, due to the emergence of the 4.0 industrial revolution, the learning theory implies that advantageous people will adapt quickly. At the same time, disadvantaged individuals face serious barriers (such as expensive tuition fees and living expenses) to improve their skills and knowledge. Regarding these two theoretical backgrounds, the income gap has become critically serious in the early stage of social development regardless of economic growth or human development, according to the Kuznets hypothesis or learning theory. Besides, the ASEAN region is an emerging economic zone which actively catches up with the global economy. Members of ASEAN are developing countries. As such, income inequality has regularly been a critical concern for their sustainable development.

In the literature, there are inconsistent conclusions about the effect of human capital on income inequality. A significant effect of human capital on reducing income inequality has been confirmed in previous studies [13–17]. Meanwhile, the opposite effects have also been reported [18,19]. However, an ambiguous effect of education expansion on income inequality has also been discussed in [20] analysis.

Based on the above considerations, the relationship between human capital and income inequality has yet to be intensively addressed in the ASEAN context, especially the theoretical link between them. As such, this study has made two contributions to the literature on human capital and income inequality. First and foremost, we develop a theoretical connection between human capital and income inequality based on two important theories, including the Kuznet theory and the learning theory. Second, we examine human capital’s short- and long-term effects on income inequality in the ASEAN context using longitudinal balanced panel data and appropriate methodology.

Following this introduction, the remainder of this paper is structured as follows. Section 2 develops a theoretical framework connecting human capital and income inequality. Section 3 presents and discusses the research method and data. Section 4 presents and discusses empirical findings, followed by concluding remarks and implications in section 5 of the paper.

## 2. Literature review

### 2.1. Theoretical background

The major focus of our research is the rising trend of inequality in the ASEAN context. Within the existing literature on inequality, various studies focus on wealth inequality for intra-country [21–23] and cross-country [24–27]. Wealth inequality is related to the difference in wealth accumulation between households or individuals. The wealth accumulating process depends on households’ and individuals’ consumption and saving behaviours. These aspects are literately different from our research objectives in which we focus on income inequality due to the differences in individual productivity with the national economic growth and development as discussed in Kuznets and the learning theories. Our research objective is closely related to macroeconomic analysis rather than microeconomic aspects of wealth inequality. Furthermore, no data are available for us to analyze, focusing on wealth inequality across countries. Regardless of the critical role of wealth inequality, we focus on explaining income inequality, especially the effect of human capital on reducing income inequality in the ASEAN region.

**Fig 1:**
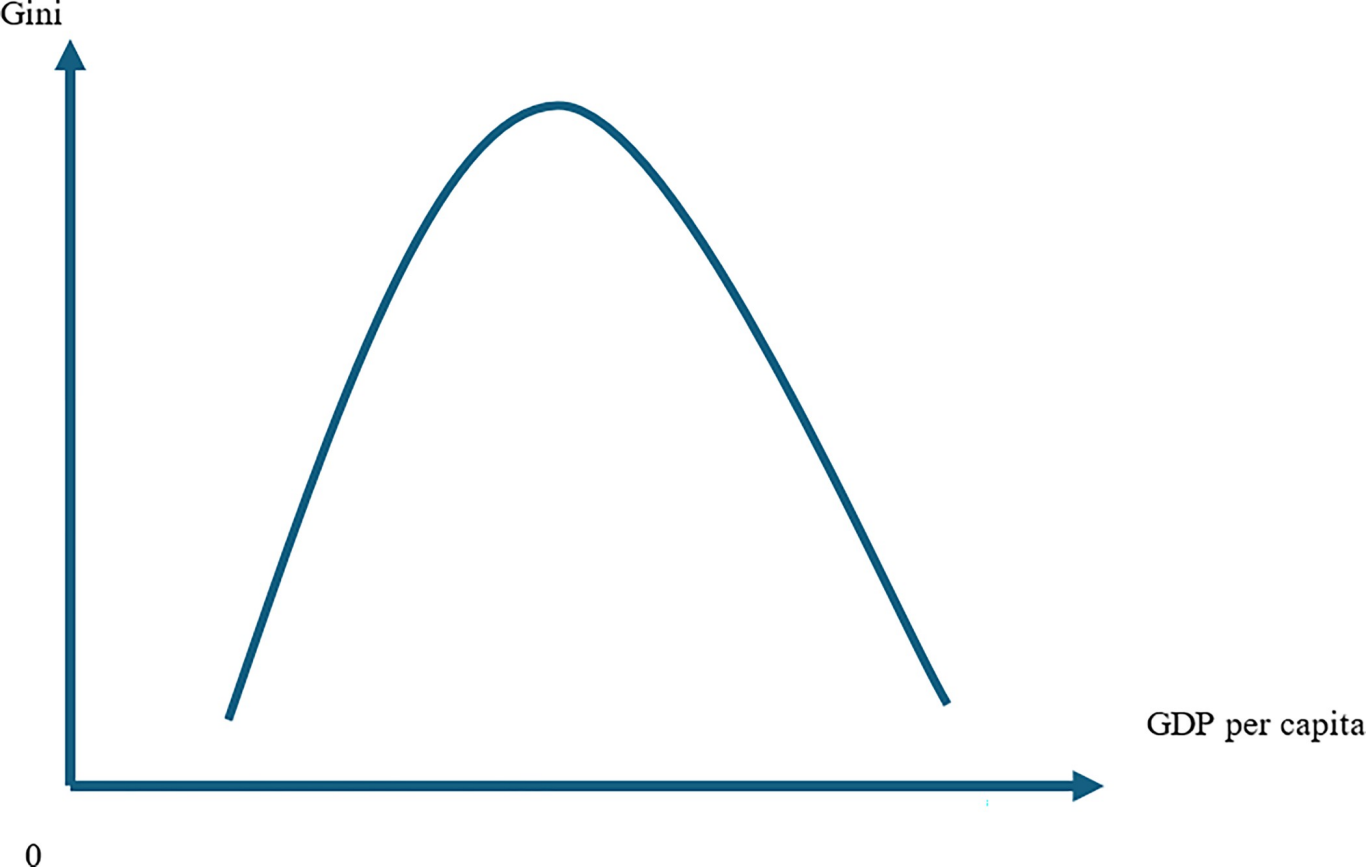
Inverted U-shape relationship between economic growth and income inequality.

To investigate the effect of human capital on income inequality in the ASEAN region, we find an undiscovered joint insight into inequality between the Kuznet theory and the learning theory. On the ground of the Kuznets hypothesis, which argues the inverted U-shaped relationship between economic growth and income inequality [4], we explore the effect of human capital on income inequality. The Kuznets hypothesis implies that at the early stage of economic growth, a country shall suffer from increased income inequality while pursuing higher income per capita. Then, at the latter stage, when income inequality reaches a certain level, it will reduce along with increased income per capita. The mechanism behind the Kuznets hypothesis is transforming the economic structure toward specialization and industrialization. In the early stage of the industrialization process, urgent demand for labour in industrial sectors led to wage differences between agriculture and industry. Fig 1 describes the inverted U-shaped relationship between income inequality and economic growth, as discussed in [4].

**Fig 2:**
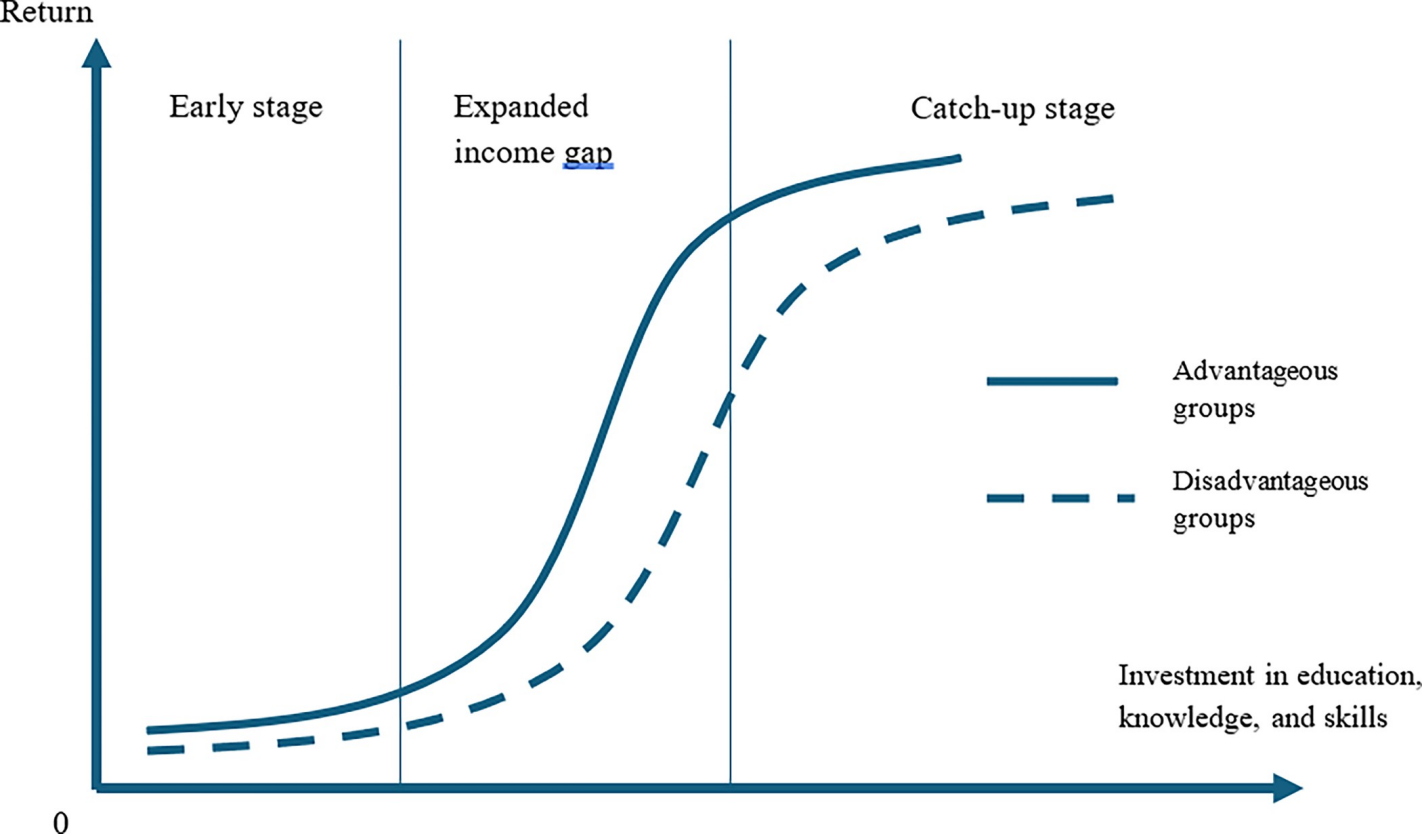
Learning theory curve (comparison between advantageous and disadvantageous groups).

The current literature presents the complex relationship between human capital and economic development. On the one hand, human capital accumulation is a consequence of economic development [28]. On the other hand, human capital also fosters economic growth [29–34]. Human capital is usually perceived as the ability of a person to generate economic value. In other words, human capital refers to human productivity within an economy. As a source of knowledge and skills, human capital can be improved through formal and informal learning (i.e., on-the-job learning or working experience). On this occasion, human capital accumulation is closely related to the “learning theory.” Among various arguments about the learning theory, [35–37] argued for a diminishing return on human capital, a consequence of the learning process, concerning investment in education. The diminishing return implies that studying performance or productivity can be illustrated throughout an upward-sloping concave curve. In other words, accumulating productivity increases steeply at the initially low level, but the amount of added outcome per additional unit of investment reduces over time. Until it reaches a certain level, the added value is critically minimal regardless of how large the investment is, as described in Fig 2.

The learning theory also describes a mechanism that implies that human capital can rapidly increase at an initially low level. However, it requires a significant investment to reach a higher level later. Besides, at the early stage of economic growth, assuming that human capital or labour productivity is low, the improvement of personal productivity favours people in the top-income group. Those people gain more advantages to improve their productivity through investing in education. With higher productivity, people in the advantageous groups gain more income and expand the income gap (the “Expand income gap” in Fig 2). However, at the latter stage, the productivity improvement in the advantaged group is minimal, while the disadvantaged group rapidly catches up (the “catch-up stage” in Fig 2). During this stage, the income gap tends to narrow because of the swift increase of productivity in the disadvantaged group and the marginal improvement in the advantaged group. This mechanism is relatively identical to the description in Kuznets’s hypothesis, in which income inequality will be expanded in the early stage of economic growth. Then, it can be narrowed down in the latter stage. The theory has also been emphasized by [20], in which the authors debated the effect of education expansion on the supply of educated labour that leads to changes in wages and results in the expansion and compression of income inequality.

Based on the above grounds, learning theory and Kuznets theory debate different perspectives of income inequality. On the one hand, Kuznets’ hypothesis directly discusses income inequality on the grounds of the equilibrium in the labour markets between agricultural and industrial sectors throughout industrialization. From the other perspective, the learning theory does not directly explain income inequality. However, the underlying learning theory describes a catching-up tendency in which individuals in disadvantageous groups improve their productivity rapidly to reach the productive level of the advantaged group. During the catching-up process, the income gap changes from widening to narrowing. As such, both theories imply that the income gap will be widened and then narrowed down in the initial and later stages of development.

While seeking the connection between the above two theories, we have found no empirical research emphasizing the similarity between Kuznets’s hypothesis and learning theory. The current paper develops a connection between human capital and income inequality based on the learning theory and Kuznets’s hypothesis. Based on the learning theory, we assume a non-linear relationship between human capital and income inequality. However, the catching-up mechanism of human capital accumulation might occur later in economic development. In other words, the tendency to catch up will happen in the long term. This expectation leads us to employ long-term estimation techniques, which will be discussed in detail in section 3 –Data and Research Methodology.

### 2.2. Empirical evidence of the effect of human capital on income inequality

Previous studies have found mixed results regarding the effect of human capital on income inequality. On the one hand, several studies have confirmed a significant positive effect of human capital on income inequality, implying that human capital accumulation reduces income inequality [13–17]. [38] argued that education improves skills and increases personal and social income, hence decreasing the dispersion of income distribution. [15,16,39] also concluded that education reduces income inequality. In contrast, other studies have found that human capital increases income inequality [18,19]. [40] argued that on-the-job training, which represents human capital, is the main factor influencing an unequal income distribution. Similarly, [41,42] stated that human capital increases income distribution. However, [20,43] presented mixed evidence on the effect of extended education on income inequality.

Recently, [14,17] found a significant effect of human capital measured by years of schooling on reducing income inequality across countries. Similarly, [44] used the human capital index and found the equalizing effect of human capital on income distribution across 30 countries. From a different aspect, [45] used the wavelet technique and confirmed the effect of human capital on reducing income inequality in Singapore. However, [46] presented the inconsistent effects of human capital on income inequality in various countries. In summary, the effect of human capital on income inequality is mixed. This complex relationship has been underexamined for the ASEAN countries. This observation warrants our study to be conducted to provide additional empirical evidence on this important link between human capital and income inequality.

## 3. Data and methodology

### 3.1. Econometric procedure

Using data from 1992 to 2018 for seven ASEAN countries, we explore the role of human capital in mitigating income inequality within the region. For long time-series panel data, we employ the mean group regressions, including the mean group (MG), pooled mean group (PMG), and the dynamic fixed effects (DFE) techniques or the fully modified ordinary least square (FMOLS) or the dynamic ordinary least square (DOLS). While the FMOLS and DOLS require all included variables in the model integrated at I (1), the mean group regressions do not. The identical assumption of stationary level is strictly adopted in the panel cointegration test [47–50]. However, a restriction of the stationary at I(1) is a critical issue for many empirical studies in which a majority of models find mixed integrated levels at both I(0) and I(1). For these cases, the mean group regressions are appropriate as mixed levels of integration among variables in the panel unit-root test reported in the next section.

First and foremost, a formal test that requires a long time-series panel analysis is the test for the presence of unit roots within the panel. In a long time-series panel, slope homogeneity and cross-sectional dependence are critical problems that can affect the unit-root test results, potentially leading to misleading decisions to conduct panel cointegration tests. Besides, testing for slope homogeneity and cross-sectional dependence allows us to use the first or the second generation of the panel unit-root tests. Accordingly, the starting point is to identify the presence of slope homogeneity and cross-sectional dependence in the panel data. If there is no slope homogeneity or cross-sectional dependence, the first generation of the unit-root test is employed. Otherwise, the second generation of unit root tests is used.

In this study, we use the cross-sectional dependence test (CD test) proposed by [51]. The test is appropriate for both balanced and unbalanced panel data. The test is illustrated as follows:

$$CD=\sqrt{\frac{2T}{N(N-1)}}\sum_{i=1}^{N-1}\sum_{j=i+1}^{N}{\hat{\sigma }}_{ij}⟶N(0,1)$$ (1)

Where N and T are cross-sectional units and time dimensions, respectively, ${\hat{\sigma }}_{ij}$ is the estimated pairwise correlation of the residuals. The CD value is assumed to be normally distributed when T and N go to infinity.

Besides the CD test, we also use the slope homogeneity test by [52], who hypothesized that b_1_ = b_2_ = … = b_n_. In other words, the estimated coefficients are homogenous across cross-sectional units. The test is described as follows:

$$S=\sum_{i=1}^{N}{\left(\beta_{i}-\beta_{WFE}\right)}^{\prime }\frac{\chi_{i}^{\prime }M_{T}\chi_{i}}{{\tilde{\sigma }}_{i}^{2}}(\beta_{i}-\beta_{WFE})$$ (2)

$$\Delta =\sqrt{N}(\frac{N^{-1}S-x}{\sqrt{2x}})$$ (3)

Where: *S* and Δ are test statistics, *β*_*i*_ is the estimated coefficients of each panel unit obtained from pooled ordinary least squares, *β*_*WFE*_ is the estimate obtained from the weighted fixed effect pooled estimator, *χ*_*i*_ is the matrix of independent variables in deviations from the mean, *M*_*T*_ is the identity matrix, ${\tilde{\sigma }}_{i}^{2}$ is an estimate of $\sigma_{i}^{2}$, x is the number of independent variables, N is number of observations.

### 3.2. Empirical model

In this study, we investigate the effect of human capital on income inequality in the ASEAN region. To explore how human capital affects income inequality, we start with the Kuznets hypothesis, which argues the inverted U-shaped relationship between economic growth and income inequality [4]. The hypothesis is described below:

$$gini_{it}=+gdppc_{it}+gdpp{c^{2}}_{it}+C_{it}+e_{it}$$ (4)

where: gini is the Gini coefficient measuring income inequality, gdppc is GDP per capita, gdppc^2^ is the square of GDP per capita, and C is the vector of control variables, e is the error term, and the subscript “t” and “i” are cross-sectional dimension and time-series dimension, respectively.

Regardless of the wide adoption of using income to calculate inequality, several studies argue the importance of wealth in inequality. There is no argument to oppose the critically important role of wealth inequality in the literature. However, wealth inequality is related to the difference in wealth accumulation between households or individuals. In this case, the wealth accumulating process depends on households’ and individuals’ consumption and saving behaviours. These aspects are literately different from our research object, in which we focus on income inequality as the result of differences in individual productivity along the economic development. Our objective is closely related to macroeconomic analysis rather than the microeconomic aspect of wealth inequality. Furthermore, there is no available database allowing us to conduct an analysis of wealth inequality across countries. Therefore, regardless of the critical role of wealth inequality, we focus on explaining income inequality in the ASEAN region.

From Eq (4), [17] argued that human capital can mitigate income inequality. Besides, human capital is also considered to have a non-linear relationship with income inequality. In particular, the relationship between human capital and income inequality is assumed to have a quadratic form. Then, the presence of human capital in Eq (5) is described as follows:

$$gini_{it}=+gdppc_{it}+gdpp{c^{2}}_{it}+hmc_{it}+hm{c^{2}}_{it}+Z_{it}+u_{it}$$ (5)

where *hmc* and *hmc*^*2*^ are human capital and squared terms of human capital, respectively. Z is a vector of control variables, and u is the error term.

However, including squared terms of GDP per capita and human capital can yield a serial correlation problem in the regression. As such, we seek a more comprehensive approach that allows us to use the original form of GDP per capita and human capital and imply the quadratic relationship between the two variables and income inequality. Among different panel-data regression techniques, the mean group regressions, which can report the short-term and long-term results simultaneously, can potentially report the non-linear relationship between the two pairs of the variables between GDP per capita–income inequality and human capital–income inequality when there are inversed results between the short and long run. However, suppose the non-linear relationship of these two pairs can exist in the short term. In that case, threshold regression allows us to capture these non-linear relations between the two threshold regions reported by the regression. Regardless of the potential advantages of the above method, we cannot assure that the quadratic form of the relationship only appears between the short run versus the long run or between the lower threshold versus the upper threshold in the mean group regression or threshold regression. As such, we employ only the original form of GDP per capita and human capital in the mean group regression and the threshold regression when we extend these two regressions by including the squared term of the two variables.

The mean group regression has three different types of regression, namely the mean group (MG), the pooled mean group (PMG), and the dynamic fixed effect (DFE). These three types of regression are distinct from others in the following perspectives. First, the MG implies heterogeneous coefficients in both short and long runs across panel units. Second, PMG only suggests a uniform coefficient across panel units in the long run. Finally, DFE is recommended if there is a homogenous coefficient across panel units in both the short and long run, which contrasts with the PMG. In our empirical context, the ASEAN-7 countries, including Indonesia, Cambodia, Malaysia, the Philippines, Singapore, Thailand, and Vietnam, share many sustainable development goals as they all belong to the same economic community (the ASEAN Economic Community–AEC). The commitment of the AEC is to develop a wealthy and sustainable economic community that reaches (i) a highly integrated and cohesive economy; (ii) competitive, innovative, and dynamic ASEAN; (iii) enhanced connectivity and sectoral cooperation; (iv) resilient, inclusive, people-oriented and people-centred ASEAN; and (v) global ASEAN. As such, these countries tend to have relatively similar development processes in the long run. In other words, the ASEAN-7 countries might have homogenous coefficients in the long run. However, in the short term, each country member has specific targets; as such, short-term coefficients shall be different across panel units. As such, among three regressions of the MG, we employ the PMG regression in our analysis. Regardless of the strong commitments among ASEAN members, there is no evidence ensuring long-term homogenous development between the ASEAN members. As such, besides the PMG regression, we also perform MG regression to confirm the robustness of our results. The PMG regression is described as follows:

$${\Delta y}_{i,t}=\theta (y_{i,t-1}+\beta x_{i,t})+\sum_{j=1}^{p}\alpha_{j}{\Delta y}_{i,t-j}+\sum_{k=1}^{q}\gamma_{k}{\Delta x}_{i,t-k}+\varepsilon_{i,t}$$

*Where*: *θ* is the error correction term; *β* is a vector of long-term coefficients; *α*_*j*_ and *γ*_*k*_ are short-term coefficients to be estimated; *x*_*i*,*t*_ is the vector of explained variables; and *ε*_*i*,*t*_ is the error term.

Finally, control variables include the share of agriculture, industry and service in GDP, development of the domestic financial system, and the globalization level in Eq (4) are drawn based on previous studies. Specifically, the share of agriculture, industry and service in GDP represents changes in the economic structure. The economic structure, including the share of agriculture, industry, and service in GDP, implies a country’s level of industrialization. These changes within the economic structure led to a transformation in the labour market across sectors, which is the underlying source of income inequality at the early stage of industrialization, as discussed in section 2. Financial development has attracted significant attention because of conflicting evidence of its effects on income inequality [53,54]. Besides, globalization has been considered a determinant of income inequality [55–58].

In the current study, we employ three sources of data, including the standardized World Income Inequality Database (SWID) by [59] (the SWID is published at https://fsolt.org/swiid/), United Nations Data, and World Development Indicators (WDI) database by the World Bank. First, [59] provided the latest updated SWID to maximize the comparability of income inequality across countries. As such, it is useful to conduct a study on income inequality using panel analysis. The database has been updated to 2023 in the latest version SWID 9.6. Second, the United Nations currently publishes statistics data from the Human Development Index (HDI) for most countries globally from 1990 to 2022. Data of HDI can be found at https://hdr.undp.org/data-center/human-development-index#/indicies/HDI. Finally, the most intensive macroeconomic data is published by the World Bank in the World Development Indicator (WDI) database providing data for other control variables in our study. WDI data is published at https://datatopics.worldbank.org/world-development-indicators/. However, to conduct the balance panel data, we can only collect data from 1992 to 2018 (all ASEAN-7 has full data on income inequality from 1992 to 2018) for all seven ASEAN nations included in our research, namely Indonesia, Laos, Malaysia, the Philippines, Singapore, Thailand, and Vietnam. Table 1 describes the descriptive statistics of our data.

**Table 1:** The descriptive statistics.

Variables	Obs.	Mean	Std. Dev.	Min	Max
Gini coefficient	189	40.057	3.188	34.200	47.500
Human development index	189	67.386	11.411	41.700	94.000
GDP per capita (Log)	189	9.206	0.968	7.611	11.496
Contribution of Industry to GDP (%)	189	34.887	7.347	16.477	48.530
Contribution of Agriculture to GDP (%)	189	14.215	9.792	0.030	51.853
Contribution of Service to GDP (%)	189	48.796	9.555	0.000	70.762
Globalization index	189	60.555	14.684	26.245	84.360
Financial development index	189	0.419	0.193	0.070	0.793

## 4. Empirical results and discussions

Table 2 presents the cross-sectional dependent and slope homogeneity tests in Panel A and Panel B, respectively. In Panel A, the null hypothesis is the cross-sectional independence of error terms between cross-sections. As the p-value is less than 0.01, we reject the null hypothesis of cross-sectional independence at 1 per cent. As such, cross-sectional dependency is exhibited in our data. In Panel B, the null hypothesis of the slope homogeneity test is that the variance of errors is constant. Since the p-value is less than 0.01, we reject the null hypothesis slope homogeneity at 1 per cent. Thus, our model is also experienced by slope heteroscedasticity.

**Table 2:** Cross-sectional dependency test and slope homogeneity test.

Test	Model	Test statistic	p_value
*Panel A*: *Cross-sectional dependency test*	re	184.901	0.000
	fe	251.873	0.000
*Panel B*: *Slope homogeneity test*		9.333	0.000

Based on the empirical results from the cross-sectional and slope homogeneity tests, as presented in Table 2, the second-generation panel unit root test should be used. Table 3 presents the results of the second-generation panel unit root tests using Pesaran Unit-root and Phillips-Perron Unit-root tests.

**Table 3:** Empirical results on the unit-root tests.

Variable	Pesaran Unit-root test		Phillips-Perron	
	Unit-root test at I(0)	Unit-root test at I(1)	Unit-root test at I(0)	Unit-root test at I(1)
*Panel A*: *Time trend is not included in the unit-root test*				
GINI	1.000	0.898	0.978	0.000
GDP per capita	0.833	0.293	0.988	0.000
GDP per capita square	0.820	0.273	0.993	0.000
HDI	0.504	0.134	0.000	0.000
Industry (% GDP)	0.956	0.000	0.900	0.000
Agriculture (%GDP)	0.783	0.005	0.000	0.000
Service (% GDP)	0.511	0.053	0.000	0.000
Globalization index	0.755	0.000	0.000	0.000
Financial development index	0.093	0.030	0.109	0.000
*Panel B*: *Time trend is included in the unit-root test*				
GINI	0.997	0.888	0.950	0.000
GDP per capita	0.890	0.976	0.421	0.000
GDP per capita square	0.908	0.972	0.589	0.000
HDI	0.829	0.310	0.398	0.000
Industry (% GDP)	0.797	0.032	0.487	0.000
Agriculture (%GDP)	0.986	0.065	0.000	0.000
Service (% GDP)	0.602	0.360	0.000	0.000
Globalization index	0.500	0.016	0.921	0.000
Financial development index	0.928	0.324	0.014	0.000

Table 4 presents the empirical results using the PMG for both the long-run and short-run effects of human capital, measured by the Gini coefficient, on income inequality for the ASEAN-6 countries. The difference between model (1) and model (2) is the inclusion of the square of GDP per capita and the square of HDI. As presented in the model (1), the results indicate that no evidence of the effect of human capital on income inequality can be established in the short run. However, human capital reduces income inequality in the long run. Our finding is different from those presented in South Africa by [60]. However, our results align with those reported in [61] studies for India. Besides, GDP per capita increases income inequality both in the long-run and short-run. Furthermore, the variation regarding economic sector structure, including industry, agriculture, and service, significantly increases income inequality in the long run.

**Table 4:** The effect of human capital on income inequality in the ASEAN countries using the pooled mean group estimation.

The dependent variable is Gini	Model 1		Model 2	
	*Long run results*	*Short-run results*	*Long run results*	*Short-run results*
Error correction term		-0.019		-0.045
		(0.029)		(0.054)
GDP per capita	4.868**	1.739*	-222.626**	73.180
	(1.899)	(1.053)	(100.395)	(75.902)
Square of GDP per capita			10.731**	-3.934
			(5.324)	(4.364)
HDI	-0.489***	-0.050	4.506*	-0.744
	(0.111)	(0.035)	(2.529)	(1.912)
Square of HDI			-0.019	0.006
			(0.016)	(0.015)
Industry (% GDP)	0.971***	-0.004	0.094	0.018**
	(0.206)	(0.011)	(0.069)	(0.007)
Agriculture (% GDP)	0.636***	0.179	-0.037	0.101
	(0.194)	(0.166)	(0.089)	(0.087)
Service (% GDP)	0.479***	0.012	0.293***	0.010
	(0.174)	(0.011)	(0.061)	(0.012)
Globalization	0.151***	0.011	0.217**	0.014
	(0.051)	(0.007)	(0.098)	(0.012)
Financial development	-2.251	-0.077	2.881	-0.154
	(2.065)	(0.435)	(3.021)	(0.184)
Constant		-0.909		40.850
		(1.302)		(49.328)
Observations	182	182	182	182

Note: Standard errors in parentheses

*, **, and *** are statistically significant at 10, 5, and 1 per cent.

Model (2) starts with model (1), adding the squared of GDP per capita. We find that human capital increases income inequality in the long run. Our empirical evidence confirms the U-shaped relationship between economic growth and income inequality in the ASEAN countries. These findings imply that income inequality may reduce as the ASEAN economies grow. However, as these economies grow further, income inequality increases in the long run. Among all the economic sectors, the service sector increases income inequality, whereas no evidence is found for the industrial and agriculture sectors. Globalization, in both models, can significantly increase income inequality in the long run. This finding is consistent with previous studies by [62].

Table 5 presents the empirical results using the mean group regression. The GINI coefficient is the dependent variable. Long-run results in Column (1) indicate that capital and financial development significantly reduce income inequality, while industry, agriculture, and services sectors expand income inequality. The globalization process increases income inequality in the ASEAN region. Besides, Kuznet’s hypothesis of the relationship between economic growth and income inequality does not exist in the long term. The Kuznets hypothesis is confirmed in Laos and Thailand. In the short run, the U-curved relationship between income inequality and economic growth is found in Indonesia and Vietnam. There is no evidence of these relationships in Malaysia, the Philippines, and Singapore. Human capital reduces income inequality in Thailand (Column 8) but increases it in Laos (Column 4). Human capital does not affect income inequality in other countries in the short run. Industry and service are found to increase income inequality in Laos and Malaysia. On the other hand, agriculture reduces income differences in Indonesia but increases income inequality in Malaysia. Globalization extends income inequality in Laos, Thailand, and Vietnam in the short run.

**Table 5:** The effect of human capital on income inequality in the ASEAN countries using the mean group estimation.

	Long run results	Short run result (All samples)	Short run result (Indonesia)	Short run result (Laos)	Short run result (Malaysia)	Short run result (The Philippines)	Short run result (Singapore)	Short run result (Thailand)	Short run result (Vietnam)
	(1)	(2)	(3)	(4)	(5)	(6)	(7)	(8)	(9)
Error Correction Term		-0.010	-0.081***	0.005**	0.001	-0.099***	-0.049***	0.200***	-0.048***
		(0.038)	(0.022)	(0.002)	(0.009)	(0.032)	(0.011)	(0.053)	(0.016)
GDP per capita	3.819	-57.760	-392.817***	13.648***	17.760	6.773	-10.110	33.628*	-73.202***
	(13.964)	(57.340)	(107.274)	(5.061)	(26.400)	(27.337)	(14.767)	(18.859)	(26.759)
GDP per capita square	-0.158	3.435	22.892***	-0.768***	-0.878	-0.377	0.444	-1.822*	4.556***
	(0.745)	(3.335)	(6.151)	(0.287)	(1.377)	(1.605)	(0.669)	(1.021)	(1.518)
HDI	-0.286***	0.003	-0.175	0.065***	-0.046	-0.076	-0.015	-0.066***	0.334
	(0.099)	(0.061)	(0.149)	(0.021)	(0.049)	(0.062)	(0.025)	(0.019)	(0.311)
Industry (% GDP)	1.426***	0.002	-0.021	0.009**	0.059***	0.000	-0.020	0.010	-0.024
	(0.312)	(0.011)	(0.025)	(0.004)	(0.019)	(0.000)	(0.021)	(0.024)	(0.018)
Agriculture (% GDP)	1.122***	0.162	-0.120*	-0.001	0.044**	-0.003	1.238	0.008	-0.031
	(0.323)	(0.180)	(0.067)	(0.003)	(0.019)	(0.027)	(1.184)	(0.017)	(0.022)
Service (% GDP)	1.037***	0.005	-0.035	0.008**	0.053***	0.039	0.000	-0.002	-0.028
	(0.314)	(0.012)	(0.030)	(0.004)	(0.020)	(0.031)	(0.023)	(0.002)	(0.018)
Globalization	0.073*	0.012	0.016	0.023***	-0.036	-0.009	-0.003	0.045***	0.047**
	(0.040)	(0.011)	(0.037)	(0.009)	(0.033)	(0.011)	(0.010)	(0.011)	(0.022)
Financial development	-5.345***	-0.243	-3.433*	0.724*	-0.381	1.239*	-0.101	-0.683**	0.936
	(1.519)	(0.596)	(1.817)	(0.414)	(0.307)	(0.746)	(0.318)	(0.291)	(0.702)

Note: Standard error in the parentheses

*, **, and *** are statistically significant at 10, 5, and 1 per cent.

Table 6 presents the empirical results on the effect of human capital on income inequality in the ASEAN countries using threshold regression. In model 1, the threshold estimates of the GDP per capita are VND 8,545.7 thousand and VND 8,212.6 thousand in model 2. For model 1, human capital is proxied by the human development index (HDI). HDI is found to significantly and positively affect income inequality in both the below and above thresholds of GDP per capita. Our empirical findings contradict those reported in [16,63], in which the empirical evidence indicated that human capital reduces income inequality. The difference between the findings could be the result of different proxies of human capital used in those studies, in which year of schooling is proxied for human capital.

**Table 6:** The effect of human capital on income inequality in the ASEAN countries using the threshold regression results.

	Model 1		Model 2	
Variables	GDP per capita < 8,545.7 USD	GDP per capita > 8,545.7 USD	GDP per capita < 8,212.6 USD	GDP per capita > 8,212.6 USD
GDP per capita	-3.234***	-2.263**	-25.174**	-3.594
	(1.144)	(1.051)	(11.980)	(12.412)
Square of GDP per capita			1.240*	0.239
			(0.697)	(0.649)
HDI	0.439***	0.151**	1.367***	-1.721***
	(0.073)	(0.075)	(0.370)	(0.525)
Square of HDI			-0.008***	0.010***
			(0.003)	(0.003)
Industry (% GDP)	-0.031	0.145**	-0.030	0.192***
	(0.028)	(0.058)	(0.032)	(0.039)
Agriculture (% GDP)	0.013	0.074	0.024	0.125*
	(0.032)	(0.084)	(0.038)	(0.066)
Service (% GDP)	-0.104***	0.166**	-0.028	0.031**
	(0.022)	(0.071)	(0.026)	(0.014)
Globalization	-0.128***	-0.148***	-0.070***	0.011
	(0.026)	(0.053)	(0.025)	(0.052)
Financial development	7.056***	-6.827***	7.833***	-1.736
	(2.523)	(1.982)	(2.197)	(1.815)
Constant	51.078***		114.445**	
	(7.492)		(45.154)	
Observations	189	189	189	189
R-squared	0.705	0.705	0.794	0.794
Number of id	7	7	7	7

Note: Standard error in the parentheses

*, **, and *** are statistically significant at 10, 5, and 1 per cent.

From the other aspect of Table 6, in model 2, the relationship between human capital and income inequality follows the inverted U-shaped relationship in the lower threshold of GDP per capita. However, this relationship follows the U-shaped relationship in the upper threshold of GDP per capita. These results from model 2 imply a possible N-shaped relationship between human capital and income inequality in the ASEAN region. Our analysis discovered the inconsistent shape of the relationship between human capital and income inequality across income regions. This finding aligns with the result from [14], in which the authors confirmed the inverted U-shape between human capital inequality and income inequality.

Based on the results of the two models in Table 6, our findings confirm the validity of the learning theory for the ASEAN countries. Income distribution tends to widen during the middle stages of economic growth and development, as depicted in Fig 2. This widening signifies transitioning from an agricultural and traditional economy to an industrial and modern society. Human capital development significantly extends the income gap between advantaged and disadvantaged groups during this stage. This gap is expected to narrow down in the later stage, following an inverted U shape. However, the U-shaped relationship between human capital and income inequality at the upper-income threshold suggests a further development stage that mirrors the learning theory process for a new transformation in the economy. This could be evidence of the income inequality trap during the social-economic development in the ASEAN region.

As presented in the model (1) of Table 6 below, the inverted U-shaped relationship between economic growth and income inequality cannot be confirmed because GDP per capita reduces income inequality across the two thresholds. However, as indicated in model 2, when the square of GDP per capita is added, the U-shaped relationship between economic growth and income inequality can be established in the lower threshold of GDP per capita. Notably, we cannot confirm the effect of economic growth on income inequality in the ASEAN countries’ upper threshold of GDP per capita. Regarding the economic structures, industry and service sectors increase income inequality in the upper threshold in models 1 and 2. These findings support the validity of the Kuznets hypothesis because changes in economic structure toward modernization and industrialization increase the income gaps by increasing labour demand in industrial and service sectors in the early stage of economic development. The ASEAN nations are especially emerging countries that have not achieved a substantial development level. As such, we find a U-shaped relationship between economic growth and income inequality in the ASEAN countries. This finding aligns with those of [64].

## 5. Conclusion and implications

The current study examines the effect of human capital on income inequality in the ASEAN countries. Specifically, we incorporate the learning theory and the Kuznets hypothesis to develop the theoretical framework for examining the effect of human capital on income inequality in this study. Besides, using the pooled mean group and the mean group regressions, we investigate the effect of human capital on income inequality in the short and long term in the ASEAN-7 countries, including Indonesia, Laos, Malaysia, the Philippines, Singapore, Thailand, and Vietnam from 1992 to 2018. We also use threshold regression to examine if there is a non-linear relationship between economic growth and income inequality and between human capital and income inequality in these ASEAN countries.

Key findings from our empirical analysis can be summarised as follows. *First*, human capital reduces income inequality in the long run, whereas the opposite effect is found in the short run. Besides, the inverted U-shaped relationship between human capital and income inequality can be established for the ASEAN countries whose income per capita is lower than USD 8.2 thousand. In contrast, the U-shaped relationship is found for countries with income per capital of more than USD 8.2 thousand. The two inverse shapes of the relationship between human capital and income inequality across different income levels indicate a potential inequality trap along socio-economic development in the ASEAN region. *Second*, we find the U-shaped relationship between economic growth and income inequality in the ASEAN-7 countries in the short- and long-term. This finding is a warning signal for the ASEAN community about the threat of increased income inequality during the process of economic growth and development in the region. However, at the country level, the U-shaped relationship is confirmed in Indonesia and Vietnam, whereas the inverted U-shaped relationship is found in Laos and Thailand. This evidence confirms the country’s unique characteristics of income inequality. Within the ASEAN community, each member exhibits particular socioeconomic conditions that can direct the equalizing impact of economic growth on income inequality in different ways. As such, studies on income inequality should consider both cross-country and intra-country levels to minimise misleading conclusions about the equalizing effect of social-economic factors. *Third*, changes in the economic structures of industry, agriculture, and services to GDP appear to extend income inequality in the ASEAN countries in the long run. This finding supports a competitive labour market in the ASEAN region, regardless of sectors, including industry, agriculture, and services. In the current economic growth and development stage, productions and services apply technological advantages that enhance the demand for skilled labour in all sectors. As a result, the income gap between skilled and unskilled labour tends to widen. *Fourth*, globalization reveals a positive effect in the short term and a negative effect in the long term on reducing income inequality in the ASEAN region. The short-term gain and long-term pain from globalization put ASEAN’s governments in a dilemma between short- and long-term development strategies. *Finally*, financial development reveals a significant effect on income inequality in the ASEAN region. Overall, the development of the financial system provides benefits in reducing income inequality in the ASEAN long-term. However, some members, such as Laos and the Philippines, as well as members whose GDP per capita is relatively low, should be aware of the expanding effect of financial development on income inequality in the short term.

Policy implications have emerged from these findings. *First*, human capital has a long-term adverse effect on income inequality. As such, ASEAN countries may need to consider national programs that support human capital accumulation via programs that improve education, health, and income in the long run. *Second*, promoting economic growth reduces income inequality in the ASEAN region. As such, policies supporting economic growth can positively reduce income inequality in the region. The governments of Indonesia and Vietnam should be aware of the U-shaped relationship between economic growth and income inequality because economic growth in these two countries may ultimately extend the income gap. *Third*, modernizing the economies by an increased contribution of industry and services sectors to GDP may lead to wider income inequality in the long run. As such, the governments in the ASEAN countries should establish specific programs or policies that support disadvantaged people, especially promoting campaigns that improve skills for unskilled labourers. *Fourth*, ASEAN members, in general, Laos, Thailand, and Vietnam, in particular, should consider parallel solutions that can extract the growth effect of globalization and reduce the impact of globalizing on increased income inequality for the long-term development. Besides, globalization might focus on specialization. Therefore, the ASEAN members should consider the key industries that specialize in each country. Then, the governments can (i) formulate and implement appropriate policies to improve labour productivity in these key industries and (ii) support other sectors to balance income equality within the society. *Finally*, ASEAN members should also consider appropriate national plans to develop their financial system to minimize the negative effects of financial development. Notably, financial inclusion can be a considerable policy in developing the national financial system and improving the allocation of financial resources within the country.

## Supporting information

S1 Data (XLSX)
